# Junctional Adhesion Molecules (JAMs): The JAM-Integrin Connection

**DOI:** 10.3390/cells7040025

**Published:** 2018-03-26

**Authors:** Daniel Kummer, Klaus Ebnet

**Affiliations:** 1 Institute-Associated Research Group: Cell Adhesion and Cell Polarity, Institute of Medical Biochemistry, ZMBE, University of Münster, Von-Esmarch-Str. 56, D-48149 Münster, Germany; daniel.kummer@uni-muenster.de; 2 Interdisciplinary Clinical Research Center (IZKF), University of Münster, D-48149 Münster, Germany; 3 Cells-In-Motion Cluster of Excellence (EXC1003-CiM), University of Münster, D-48149 Münster, Germany

**Keywords:** cis-interaction, integrin, junctional adhesion molecule (JAM), JAM-A, trans-interaction, leukocyte adhesion, signaling complex, tetraspanin, tetraspanin-enriched microdomain

## Abstract

Junctional adhesion molecules (JAMs) are cell surface adhesion receptors of the immunoglobulin superfamily. JAMs are involved in a variety of biological processes both in the adult organism but also during development. These include processes such as inflammation, angiogenesis, hemostasis, or epithelial barrier formation, but also developmental processes such as hematopoiesis, germ cell development, and development of the nervous system. Several of these functions of JAMs depend on a physical and functional interaction with integrins. The JAM – integrin interactions in trans regulate cell-cell adhesion, their interactions in cis regulate signaling processes originating at the cell surface. The JAM – integrin interaction can regulate the function of the JAM as well as the function of the integrin. Beyond the physical interaction with integrins, JAMs can regulate integrin function through intracellular signaling indicating an additional level of JAM – integrin cross-talk. In this review, we describe the various levels of the functional interplay between JAMs and integrins and the role of this interplay during different physiological processes.

## 1. Introduction

Junctional adhesion molecules (JAMs) belong to the immunoglobulin superfamily (IgSF) of cell adhesion receptors. The founding member JAM-A was originally identified in platelets [1]. Molecular cloning revealed a much broader tissue distribution of JAM-A [2,3,4,5,6] and led to the identification of JAM-A-related proteins such as JAM-B [7,8,9,10] and JAM-C [9,11,12], as well as of the more distantly related JAM4 [13] and JAM-L [14]. It is now clear that JAMs are expressed by a large variety of cell types and tissues, including epithelial cells and endothelial cells, leukocytes, cells of the male reproductive system, cells of the central and peripheral nervous systems, and fibroblasts [15,16,17]. 

One feature of JAMs is their ability to undergo homophilic interactions with JAMs expressed by the connected cell. However, JAMs can also undergo heterophilic interactions, and these interactions can occur both in cis and in trans [18]. Cis interactions are referred to as lateral associations with other integral membrane proteins expressed by the same cell, which can be mediated by the extracellular domains or by the cytoplasmic domains of the partners. Trans interactions are referred to as interactions which involve the extracellular domains of two proteins that are expressed by two different cells [19,20]. More recent findings revealed members of the integrin superfamily as major partners of JAM heterophilic interactions in both cis and trans. Integrins are heterodimeric cell surface proteins which are expressed by all multicellular organisms [21,22] and which mediate cell-matrix as cell-cell interactions. The adhesive activity of integrins is regulatable by cell surface receptor-initiated signals which trigger a switch from an inactive to an active conformation able to interact with ligands [21,23]. On the other hand, ligand occupation of integrins activates intracellular signaling cascades through a diverse set of proteins associated with the cytoplasmic tails of integrins [24]. The identified physical interactions of JAMs with integrins as well as the functional crosstalk that occurs between JAMs and integrins suggest an important role of JAMs in influencing integrin-mediated processes and vice versa. In this review, article, we summarize the recent observations which indicate a mutual functional regulation of JAMs and integrins.

## 2. JAM-Integrin Interactions in Trans

**Figure 1:**
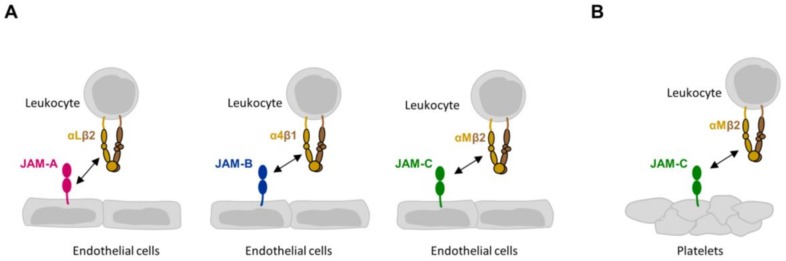
Heterophilic JAM-integrin interactions in trans. (**A**) Trans interactions between JAMs expressed by endothelial cells and integrins on leukocytes. Note that the interaction of αLβ2 integrin with JAM-A involves the membrane-proximal Ig domain of JAM-A. The interactions between JAM-B and α4β1 integrin, and between JAM-C and αMβ2 integrin have not been mapped in detail. (**B**) Trans interaction between JAM-C expressed by platelets and αMβ2 integrin on leukocytes.

All trans-interactions of JAMs with integrins identified so far occur during inflammation and mediate the transient interactions between leukocytes and various target cells such as epithelial cells, endothelial cells, or platelets [15,17,25,26] (Figure 1). Leukocytes can express either a JAM family member or a member of the integrin family, and the activity of both the JAM molecule and the integrin molecule can be regulated by exogenous factors. This ensures fine tuning of the cellular interactions, which is necessary for leukocytes to extravasate and migrate through tissues to reach the sites of inflammation.

During inflammation, leukocytes are recruited to sites of injury through a multi-step process that involves leukocyte tethering to and rolling along the luminal endothelial cell surface, the activation of leukocyte integrins which triggers firm adhesion of the leukocyte to the endothelium, and finally the transendothelial migration of leukocyte and their migration within the tissue [27,28]. The presence of multiple steps in this process helps to prevent unwanted tissue infiltration of leukocytes and also to guide specific immune cells to secondary lymphoid organs [29]. During all steps, from the initial tethering until their release into the connective tissue, leukocytes are in intimate contact with endothelial cells. The JAM-integrin-mediated interactions in trans seem to contribute to several steps during this process.

### 2.1. JAM-A and αLβ2 Integrin

αLβ2 integrin (lymphocyte function-associated antigen 1 (LFA-1), CD11a/CD18) is a leukocyte-specific integrin that is predominantly expressed by T cells [30]. Similar to other integrins, αLβ2 integrin exists in a state of low affinity. Signals through agonists such as chemokines or selectin ligands switch the integrin conformation to a high affinity state that allows αLβ2 integrin to interact in trans with its natural ligands such as intercellular cell adhesion molecules (ICAMs) [23], an interaction that contributes to the firm adhesion of activated T cells to the endothelium. 

A screen searching for cytoplasmic binding partners of the αL integrin chain identified JAM-A as ligand for αLβ2 integrin [31]. The interaction, however, occurs predominantly in trans through the extracellular domains of the two proteins. The trans interaction of αLβ2 with JAM-A is strong enough to mediate cell adhesion in static adhesion assays on immobilized ligands [31], which suggests that the heterophilic JAM-A – αLβ2 integrin is stronger than the homophilic JAM-A – JAM-A interaction, the latter of which cannot be demonstrated under analogous assay conditions [8]. The αLβ2 – JAM-A integrin interaction cooperates with the αLβ2 – ICAM-1 interaction to mediate T cell adhesion to inflamed endothelium in an additive manner. Interestingly, when JAM-A function is blocked, T cells are also impaired in undergoing cell spreading and transendothelial migration, suggesting that the αLβ2 – JAM-A integrin interaction feeds back into leukocytes to regulate post-adhesive events [31]. As an additional evidence for a functional role of the αLβ2 integrin – JAM-A interaction in vivo, the inhibition of this interaction attenuates the trafficking of leukocytes into the brain after hypoxia-reperfusion-triggered brain inflammation [32].

Endothelial JAM-A shows a unique behavior that accounts for the ability of JAM-A to contribute to a process that occurs at the apical membrane domain. JAM-A as a classical cell-cell adhesion molecule is enriched at cell-cell contacts of epithelial cells and endothelial cells [2]. However, in endothelial cells pro-inflammatory stimuli, such as a combination of TNF-α and IFNγ or the chemokine CCL2, trigger a redistribution of JAM-A from cell-cell junctions to the apical membrane domain, thus making JAM-A available at the luminal surface for leukocyte interactions [33,34].

The biochemical interaction of JAM-A with αLβ2 integrin is mediated by the membrane-proximal Ig-like domain (D1 domain) of JAM-A and the inserted domain (I domain) of the αLβ2 integrin, which is present in the αL subunit [31,35] and which is the principal ligand-binding domain for the trans-interaction with ICAM-1 [30]. The interaction with the D1 domain of JAM-A has important implications. The dimerization of JAM-A in cis and trans-homophilic interactions are both mediated by the membrane-distal (D1) domain of JAM-A [20,36,37]. Importantly, however, the D2 domain contributes to the strength of the trans-homophilic interaction, probably by stabilizing the JAM-A cis dimer [38]. Moreover, the binding of αLβ2 integrin to the D2 domain of JAM-A destabilizes the trans-homophilic interaction of JAM-A [38]. The αLβ2 integrin-mediated binding of leukocytes to endothelial cell-expressed JAM-A therefore most likely influences the state of dimerization of JAM-A. Since recent evidence suggests a role of JAM-A monomers in endothelial signaling through cis-heterophilic interactions with tetraspanins and integrins [39] (see also above), any changes in the state of dimerization could result in altered endothelial signaling, which is required for the opening of endothelial cell-cell contacts during leukocyte extravasation [40]. These JAM-A-triggered signaling events could be initiated at the apical domain during leukocyte firm adhesion and further sustained during leukocyte transendothelial migration, when migrating leukocyte may disrupt the trans-homophilic interaction of JAM-A between adjacent endothelial cells. Indeed, αLβ2 integrin and JAM-A co-localize in ring-like structures around the sites of penetration of transmigrating leukocytes [41].

### 2.2. JAM-C and αMβ2 Integrin

αMβ2 integrin (macrophage antigen 1 (MAC-1), CD11b/CD18) is the second leukocyte-specific integrin which interacts in trans with a JAM family member. αMβ2 integrin is predominantly expressed by monocytes/macrophages and neutrophils but also by dendritic cells (DC) [23]. Its major ligands are the inactivated complement component C3b (iC3b), which acts as opsonin for pathogens, as well as fibrinogen and heparin [23]. αMβ2 integrin thus helps phagocytes to engulf bacterial pathogens and to boost the inflammatory response by the fibrinogen and/or heparin binding-triggered release of cytokines. 

αMβ2 integrin interacts in trans with JAM-C [42,43]. Similar to the JAM-A – αLβ2 integrin interaction, the JAM-C – αMβ2 integrin interaction is stronger than the homophilic JAM-C – JAM-C interaction, as the former interaction supports cell adhesion on immobilized ligands whereas the latter one does not [42]. Since JAM-C is expressed by platelets and T lymphocytes (in humans) as well as by endothelial cells (in humans and mice) [17], the JAM-C – αMβ2 interaction most likely regulates various aspects of the inflammatory response. Strong evidence has been provided for a role in neutrophil adhesion to platelets which is mediated by αMβ2 integrin on neutrophils and JAM-C on platelets [42], and which helps to recruit neutrophils to surface-adherent platelets and assists their transmigration across the vessel wall [44]. DC adhesion to platelet deposits has been found to be mediated by αMβ2 integrin on DCs and JAM-C on platelets, and this interaction seems to activate DCs resulting in increased DC activities such as phagocytic activity and cytokine release [45]. Endothelial cell-expressed JAM-C is localized at intercellular cell-cell junctions through a trans-homophilic interaction [43], suggesting that the interaction with leukocyte αMβ2 integrin occurs during leukocyte transendothelial migration. Interestingly, JAM-C interacts with endothelial cell-expressed JAM-B in a trans-heterophilic manner, and this interaction is stronger than the homophilic JAM-C – JAM-C interaction [43]. Thus, JAM-C expressed by transmigrating T lymphocytes could disrupt the heterophilic JAM-B – JAM-C interaction thereby liberating JAM-C from endothelial cell-cell junctions and making it accessible at the luminal surface to support leukocyte adhesion to the inflamed endothelium. In support of this, JAM-C antibodies result in a relocalization of JAM-C [43,46], and a redistribution of JAM-C to the non-junctional membranes was observed during ischemia/reperfusion injury [47]. A role for JAM-C in regulating the inflammatory response has been demonstrated in various pathophysiological settings [46,47,48,49,50,51,52,53,54,55]. 

The biochemical interaction of JAM-C with αMβ2 integrin has not been characterized in detail. Most likely, it involves the I-domain of the αMβ2 integrin [42]. The interaction interface in JAM-C has not been determined yet. If the αMβ2 integrin binding to JAM-C disrupts the JAM-C dimer as described for the JAM-A – αLβ2 integrin interaction [38] is unclear.

### 2.3. JAM-C and αXβ2 Integrin

The leukocyte specific αXβ2 integrin (p150/95, CD11c/CD18) is also a ligand for JAM-C, and this interaction also supports cell adhesion [42]. The αXβ2 integrin has a similar expression profile to αMβ2 integrin and the two integrins share most of their ligands [23]. However, the JAM-C – αXβ2 interaction seems to play a less prominent role than the JAM-C – αMβ2 interaction, since several JAM-C – β2 integrin mediated functions such as neutrophil adhesion to platelets or neutrophil adhesion to purified JAM-C are blocked by anti-αMβ2 antibodies but not by anti-αXβ2 antibodies [42].

### 2.4. JAM-B and α4β1 Integrin

The α4β1 integrin (very late antigen (VLA)-4, CD49d/CD29) is not leukocyte-specific but is expressed at high levels by various leukocyte subsets such as lymphocytes or monocytes [23,56]. It is primarily involved in the initial steps of the leukocyte adhesion cascade, i.e., in the tethering/rolling of leukocytes and their firm adhesion to the endothelial surface [57].

α4β1 integrin interacts in trans with JAM-B [58]. This interaction is enhanced upon integrin activation and requires the co-expression of JAM-C on the leukocyte surface [58]. This unusual finding could suggest that JAM-C is required for α4β1 integrin to adopt its fully active conformation allowing its interaction with JAM-B, or alternatively, that the JAM-C – JAM-B interaction contributes to the interaction between the two cell types, perhaps after an α4β1 integrin conformation-dependent release of monomeric JAM-C from the integrin, allowing its dimerization and trans-heterophilic interaction with JAM-B. A similar mechanism has been described for leukocyte-specific JAML, which is retained in a complex with α4β1 integrin as adhesion-incompetent monomer, and which is released from this complex upon integrin activation to undergo cis-dimerization followed by trans-heterophilic interaction with CAR [59]. Consistent, however, with a role for the heterophilic α4β1 integrin – JAM-B interaction is the observation that T cell rolling and adhesion to immobilized JAM-B is blocked by anti α4β1 integrin antibodies but not by anti JAM-C antibodies [60]. 

The interaction of α4β1 integrin on leukocytes to endothelial JAM-B has been shown to require the membrane-distal Ig domain of JAM-B [58]. As a deletion of this domain most likely prevents cis-dimerization which is mediated by R_59_L_60_E_61_ motif present in the membrane-distal Ig-like domain, an interaction with the membrane-proximal Ig domain through a motif that depends on cis-dimer formation cannot be excluded.

## 3. JAM-Integrin Interactions in Cis

**Figure 2:**
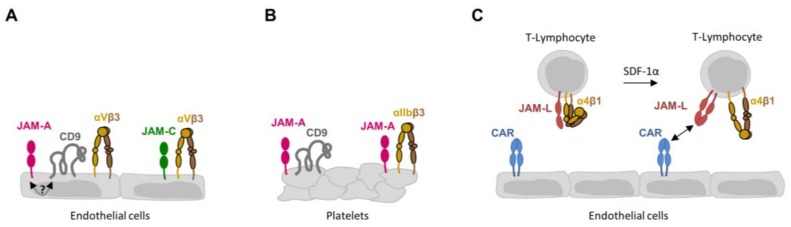
Heterophilic JAM-integrin interactions in cis. (**A**) Cis interactions between JAMs and αVβ3 integrin in endothelial cells. The interaction between JAM-A and αVβ3 integrin is mediated by tetraspanin CD9. The interaction between JAM-A and CD9 requires the PDZ domain binding motive of JAM-A and is therefore most likely mediated by an unidentified cytoplasmic protein. (**B**) Cis interaction between JAM-A and αIIbβ3 integrin in platelets. Similar to endothelial cells, JAM-A interacts with both CD9 and the β3 integrin (αVβ3 integrin in endothelial cells, αIIbβ3 integrin in platelets), suggesting the JAM-A – αIIbβ3 integrin is mediated by CD9. (**C**): Cis interaction between JAM-L and α4β1 integrin in T-lymphocytes. Note that in unstimulated T-lymphocytes, α4β1 integrin is associated with monomeric JAM-L. Stimulation by SDF-1α releases JAM-L monomers from α4β1 integrin, allowing cis dimer formation followed by trans interaction with CAR on endothelial cells.

*Cis* interactions of JAMs with integrins have so far been found to exist on endothelial cells, leukocytes, and platelets [17] (Figure 2). It is thus not surprising that these lateral associations are involved in processes such as angiogenesis, vascular permeability, hemostasis, or inflammation. It is common to all lateral associations between JAMs and integrins that these interactions are coupled to intracellular signaling cascades, in which the JAM family member can act both as an upstream initiator and as a downstream recipient of a signaling cascade.

### 3.1. JAM-A and JAM-C Interact with αVβ3 Integrin in Endothelial Cells

αVβ3 integrin and αVβ5 integrin are the two vitronectin receptors expressed by endothelial cells [24]. Although both bind to vitronectin, they promote distinct growth factor-dependent signaling pathways: mitogen-activated kinase (MAPK)—extracellular signal-regulated kinase (ERK) pathway stimulation by bFGF requires αVβ3 integrin whereas stimulation by VEGF requires αVβ5 integrin [61]. 

In endothelial cells JAM-A interacts with αVβ3 integrin but not with αVβ5 integrin [39,62,63]. In accordance with this selective interaction of JAM-A with αVβ3 integrin, JAM-A-regulated migration on vitronectin can be blocked with αVβ3 integrin antagonists or αVβ3 integrin-specific antibodies but not with αVβ5 integrin antibodies [63]. In addition, depletion of JAM-A prevents bFGF- but not VEGF-triggered activation of the MAPK-ERK pathway [39]. These observations thus indicate a role of JAM-A specifically in the bFGF-stimulated MAPK-ERK pathway activation, which is most likely mediated through its association with αVβ3 integrin. The mechanism underlying the role of JAM-A in bFGF-triggered MAPK-ERK activation is still unclear. JAM-A’s association with αVβ3 integrin is mediated by the tetraspanin family member CD9 [39]. As observed for JAM-A, CD9 is required for bFGF- but not VEGF-triggered activation of the MAPK-ERK pathway, strongly suggesting that CD9 forms an essential link between JAM-A and αVβ3 integrin. bFGF triggers the dissociation of JAM-A from CD9 and αVβ3 integrin [39,62]. Since JAM-A associated with CD9 and αVβ3 integrin exists predominantly as monomer, it is conceivable that a release of monomeric JAM-A from the complex results in the formation of a signaling-competent and active JAM-A dimer which initiates signaling from the membrane [39]. The functional relevance of the JAM-A – αVβ3 integrin association has been confirmed in mice. JAM-A-deficient mice fail to mount an angiogenic response both in aortic ring sprouting assays and Matrigel plug assays in response to bFGF [64]. Of note, JAM-A has also been found to regulate wound healing-associated neoangiogenesis by negatively regulating VEGF signaling [65]. The mechanism underlying this negative regulatory function is still unclear.

Besides JAM-A, JAM-C has also been described to interact with αVβ3 integrin in endothelial cells [66]. Although the nature of the interaction has not been examined in detail, it is likely that the association occurs in cis. The functional relevance of the JAM-C – αVβ3 integrin association, however, is unclear. The junctional levels of the β1- and β3 integrin chains as well as β1- or β3-integrin-mediated adhesion to various extracellular matrix components negatively correlate with JAM-C expression levels. Similarly, the levels of active Rap1 negatively correlate with JAM-C expression [66]. JAM-C has been implicated in vascular permeability by regulating the levels of vascular-endothelial cadherin (VE-cadherin) at endothelial cell-to-cell contacts through Rap1 [67]. If this activity of JAM-C depends on its association with αVβ3 integrin has not been studied yet.

### 3.2. JAM-A and αIIbβ3 Integrin in Platelets

Integrin αIIbβ3 is the major integrin expressed at the surface of platelets and is essential for hemostasis by mediating platelet aggregation and platelet spreading [68]. Activated integrin αIIbβ3 binds extracellular ligands such as fibrinogen, resulting in integrin αIIbβ3 microclustering and assembly of intracellular signaling complexes involving tyrosine kinases c-Src and Syk and subsequent platelet activation [69]. 

In platelets, JAM-A interacts with αIIbβ3 integrin [70,71]. This association is likely to be indirect and mediated by CD9, since similar to what was observed in endothelial cells, CD9 has also been identified to be associated with JAM-A in platelets [70]. However, as opposed to endothelial cells in which JAM-A positively regulates intracellular signaling in response to bFGF, JAM-A prevents intracellular signaling in platelets [71,72]. This function of JAM-A as negative regulator of intracellular signaling is mediated by c-Src kinase (Csk), which binds to the JAM-A cytoplasmic domain in resting platelets [71] thereby inhibiting activation of αIIbβ3-associated c-Src in the absence of agonists. Platelet activation with thrombin, fibrinogen, or ADP results in the release of JAM-A from αIIbβ3 integrin and at the same time in JAM-A dephosphorylation by protein phosphatase PTPN1, resulting in the dissociation of Csk from the complex thus allowing activation of αIIbβ3-associated c-Src [71,72]. This mechanism is highly relevant in vivo as indicated by a hyperreactivity of platelets in JAM-A-deficient mice, which is accompanied by enhanced thrombus formation and increased predisposition to atherosclerosis [72,73].

### 3.3. JAM-L and α4β1 Integrin in Leukocytes

As mentioned before, α4β1 integrin is expressed at high levels by various leukocyte subsets such as lymphocytes and monocytes and is involved in the tethering/rolling of leukocytes and their firm adhesion to the endothelial surface [57]. JAM-L is a JAM family member that is less closely related to JAM-A, -B, and -C than the three JAMs to each other [14]. It interacts in trans with another distantly related JAM family, i.e., coxsackie and adenovirus receptor (CAR) [74]. The JAM-L – CAR trans-heterophilic interaction has so far been described to mediate the interaction of leukocytes with epithelial cells, endothelial cells, and keratinocytes [14,59,74,75,76], 

JAM-L is expressed by various types of leukocyte including neutrophils, monocytes and specific subsets of T cells [59,75]. Interestingly, in leukocytes such as neutrophils which do not express α4β1 integrin, JAM-L is constitutively active, whereas in α4β1 integrin-expressing leukocytes such as monocytes or T lymphocytes, JAM-L trans-interaction with CAR requires integrin activation [59]. In these cells, JAM-L is associated with α4β1 integrin as monomer. Integrin stimulation by the chemokine SDF-1α releases JAM-L from α4β1 integrin allowing the formation of cis dimers. As also shown for JAM-A, -B, and -C, the cis dimeration is the prerequisite for interactions in trans. In the case of JAM-L, the dimer interacts in trans with CAR to strengthen the interaction between T lymphocytes and endothelial cells [59]. It is likely that monomeric JAM-L has a high affinity to the inactive integrin and that this affinity is strongly reduced upon integrin activation, which is accompanied by an upright conformation [23]. The JAM-L – α4β1 integrin association provides a unique example of a regulation of JAM binding activity by a lateral association with an integrin family member.

## 4. JAM-Integrin Crosstalk

Apart from interacting directly or indirectly with integrins JAMs can also regulate the levels of integrin expression. In SK-CO15 colonic epithelial cells and in MCF7 breast carcinoma cells, the protein levels of β1 integrin are reduced after depletion of JAM-A [77,78,79]. Consequently, JAM-A knockdown cells fail to spread, adhere, and migrate properly on ECM proteins in a β1 integrin-dependent manner. The regulation of β1 integrin expression levels has been attributed to the ability of JAM-A to activate Rap1 by recruiting Afadin, a scaffolding protein for Rap1, and PDZ-GEF2, a guanine nucleotide exchange factor for Rap1 [78,80]. The close spatial proximity generated by JAM-A allows the functional interaction of PDZ-GEF2 with Rap1. In neutrophils, the absence of JAM-A results in a reduced ability to migrate through the interstitial tissue [81]. Interestingly, however, the reduced migratory capacity of JAM-A-deficient neutrophils is not caused by reduced β1 integrin expression but is due to an impaired β1 integrin recycling. At the molecular level, JAM-A is recruited to cell surface-expressed β1 integrins and through an as yet unknown mechanism induces their internalization, which is a prerequisite for efficient uropod retraction during migration [81]. A role in the regulation of integrin-mediated processes such as cell adhesion and cell migration has also been identified for JAM-C in the epithelia-derived tumor cell line KLN205 [82]. Ectopic expression of a phosphorylation-deficient mutant of JAM-C decreases cell spreading but increases cell adhesion and migration. This function of JAM-C is mediated through phosphorylation at Ser281, which regulates the activities of β1 and β3 integrins in a reciprocal manner [82]. In contrast to the regulation of β1 integrin expression in SK-CO15 colonic epithelial cells and the β1 integrin surface exposure in neutrophils by JAM-A, JAM-C regulates the binding activities of β1 and β3 integrins in these cells [82]. Together, these findings indicate an intimate crosstalk between JAMs and integrins and highlight the versatility of JAMs in the regulation of integrin-mediated processes.

## 5. Concluding Remarks

Many functions of JAMs depend on a physical and/or functional interaction with integrins. Physical interaction in trans involve integrins on leukocytes and regulate the adhesion of leukocytes to endothelial cells or platelets. Physical interactions in cis involve β3 integrins in endothelial cells and in platelets and most likely regulate integrin-mediated signaling processes, as shown in platelets where JAM-A regulates the activity of αIIbβ3 integrin-associated c-Src [71]. The cis-interaction with integrins are indirect and mediated by tetraspanins [39,70], suggesting that tetraspanins play a major role in connecting JAMs to integrins and allowing their functional interaction. In general, JAM-integrin interactions can regulate the function of each partner. For example, the integrin can regulate the adhesive activity of a JAM family member by keeping it in a non-functional, monomeric state [59]. Alternatively, the JAM family member can regulate the activity of the integrin by stimulating integrin internalization and cell surface turnover [81], or by regulating small GTPases upstream of integrin expression and activity [83]. The JAM-integrin trans interactions probably signal back into the two cell types involved, as for example indicated by a reduced ability of T lymphocytes to spread and transmigrate after inhibition of JAM-A function [31], or by the disruption of the JAM-A dimer upon αLβ2 integrin interaction, which most likely affects the signaling activity of JAM-A [38]. It is to be expected that more aspects of the JAM-integrin connection will be discovered in the future.
